# Etymologia: Ebola

**DOI:** 10.3201/eid2111.ET2111

**Published:** 2015-11

**Keywords:** etymologia, Ebola, Ebola virus, filovirus, viruses, Myriam Louise Ecran, Frederick Murphy, Joel Breman, Karl Johnson, Zaire

## Ebola [ebʹo-lə]

**Figure 1:**
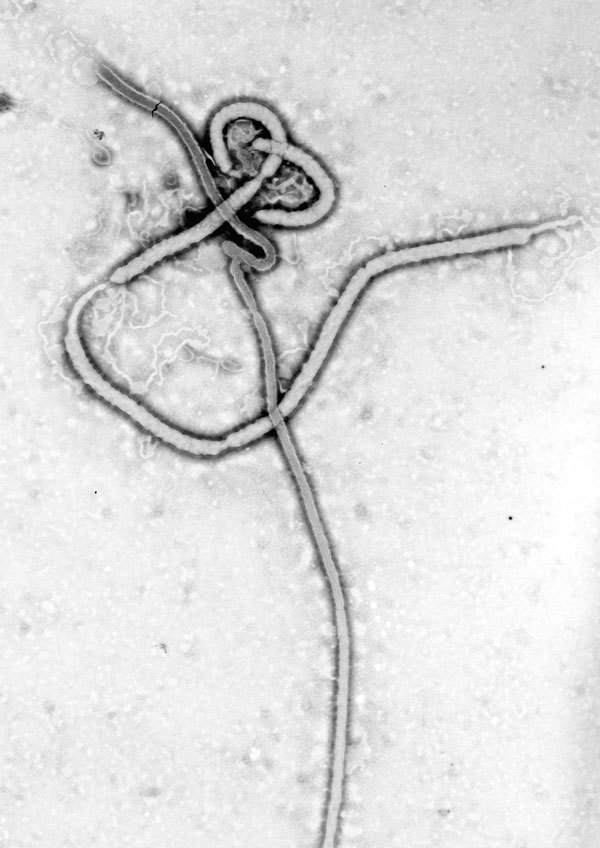
Taken by Frederick Murphy at CDC, this iconic transmission electron micrograph shows the filamentous shape of the Ebola virus. On October 13, 1976, Murphy captured this image and, along with Karl Johnson and Patricia Webb, carried the printed negative, dripping wet, directly to CDC Director David Sencer. At the time, they were among the only persons in the world to have seen this “dark beauty” (F.A. Murphy, pers. comm.).

**Figure 2:**
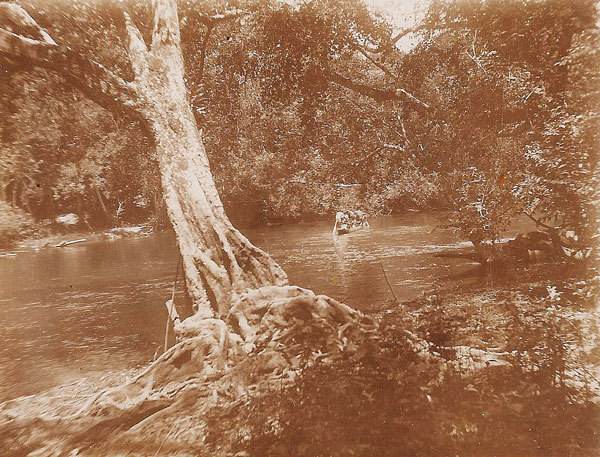
Ebola River, ca. 1932. Photo courtesy Pierre Rollin.

Ebola virus, discovered in 1976 during an outbreak in Zaire (now Democratic Republic of the Congo), was first isolated from Myriam Louise Ecran, a 42-year-old Belgian nursing sister working at the Yambuku Mission Hospital who died caring for people with this unknown disease. When the international commission considered the name “Yambuku virus,” Karl Johnson and Joel Breman noted that naming the Lassa virus after the Nigerian village where it was discovered brought stigma to the community. Johnson suggested naming the virus after a nearby river, and the rest of the commission agreed (Figure 1).. The Belgian name for the river, *l’Ebola*, is actually a corruption of the indigenous Ngbandi name *Legbala*, meaning “white water” or “pure water” (J.G. Breman, L.E. Chapman, F.A. Murphy, P.E. Rollin, pers. comm.) (Figure 2).

The Ebola virus, originally described as “Marburg like,” was determined to be a related filovirus (from the Latin *filum*, “thread”), named for the elongated, flexible shape. The virus was first described in 3 back-to-back articles in The Lancet in 1977.

## References

[1] Bowen ET, Lloyd G, Harris WJ, Platt GS, Baskerville A, Vella EE. Viral haemorrhagic fever in southern Sudan and northern Zaire. Preliminary studies on the aetiological agent. Lancet. 1977;1:571–3 and. 10.1016/S0140-6736(77)92001-3 65662

[2] DelViscio J. A witness to Ebola’s discovery. The New York Times. 2014 Aug 9 [cited 2015 Aug 4]. http://www.nytimes.com/2014/08/08/science/a-witness-to-ebolas-discovery.html.

[3] Johnson KM, Lange JV, Webb PA, Murphy FA. Isolation and partial characterization of a new virus causing acute haemorrhagic fever in Zaire. Lancet. 1977;1:569–71 and. 10.1016/S0140-6736(77)92000-1 65661

[4] Pattyn S, Jacob W, van der Groen G, Piot P, Courteille G. Isolation of Marburg-like virus from a case of haemorrhagic fever in Zaire. Lancet. 1977;1:573–4 and. 10.1016/S0140-6736(77)92002-5 65663

[5] Tanghe B, Vangele A. The high Ebola region. Historical notes (1890–1900) [in French]. Aequatoria. 1939;2:61–5.

[6] Wordsworth D. How Ebola got its name. The Spectator. 2014 Oct 25 [cited 2015 Aug 4]. http://www.spectator.co.uk/life/mind-your-language/9349662/how-ebola-got-its-name/

